# Isolation and Characterisation of Electrogenic Bacteria from Mud Samples

**DOI:** 10.3390/microorganisms11030781

**Published:** 2023-03-17

**Authors:** György Schneider, Dorina Pásztor, Péter Szabó, László Kőrösi, Nandyala Siva Kishan, Penmetsa Appala Rama Krishna Raju, Rajnish Kaur Calay

**Affiliations:** 1 Department of Medical Microbiology and Immunology, Medical School, University of Pécs, Szigeti Str. 12, H-7624 Pécs, Hungary; 2 Department of Geology and Meteorology, Faculty of Sciences, University of Pécs, Ifjúság Str. 6, H-7624 Pécs, Hungary; 3 Research Institute for Viticulture and Oenology, University of Pécs, Pázmány P. u. 4, H-7634 Pécs, Hungary; 4 Centre for Research and Development, SRKR Engineering College, SRKR Marg, China Amiram, Bhimavaram 534204, India; 5 Department of Civil Engineering, SRKR Engineering College, SRKR Marg, China Amiram, Bhimavaram 534024, India; 6 Institute for Building Energy and Materials Technology, Narvik Campus, UiT Norway’s Arctic University, 8514 Narvik, Norway

**Keywords:** electrogenic bacteria, identification, mud, biofilm, abiotic surface, carbon tissue, enzymatic assays

## Abstract

To develop efficient microbial fuel cell systems for green energy production using different waste products, establishing characterised bacterial consortia is necessary. In this study, bacteria with electrogenic potentials were isolated from mud samples and examined to determine biofilm-formation capacities and macromolecule degradation. Matrix-assisted laser desorption/ionization time-of-flight mass spectrometry identifications have revealed that isolates represented 18 known and 4 unknown genuses. They all had the capacities to reduce the Reactive Black 5 stain in the agar medium, and 48 of them were positive in the wolfram nanorod reduction assay. The isolates formed biofilm to different extents on the surfaces of both adhesive and non-adhesive 96-well polystyrene plates and glass. Scanning electron microscopy images revealed the different adhesion potentials of isolates to the surface of carbon tissue fibres. Eight of them (15%) were able to form massive amounts of biofilm in three days at 23 °C. A total of 70% of the isolates produced proteases, while lipase and amylase production was lower, at 38% and 27% respectively. All of the macromolecule-degrading enzymes were produced by 11 isolates, and two isolates of them had the capacity to form a strong biofilm on the carbon tissue one of the most used anodic materials in MFC systems. This study discusses the potential of the isolates for future MFC development applications.

## 1. Introduction

Electrogenic bacteria are a group of microorganisms that can transfer electrons across the cell envelope onto different electron acceptors, such as electrodes and minerals, or even to another bacteria. The process is based on the electrochemical interactions between microbes and electrodes [1,2]. For this process to function, a potential microbe must have proper reducing power and a cellular mechanism through which the electrons can be transferred from the living organism onto the abiotic surface [3]. This electron transfer process can occur on the anodic surface of microbial fuel cells (MFC) and can be exploited for electron production. Because of this, MFCs have begun to receive considerable attention as potential sustainable energy production units that are able to convert organic waste biomass to electricity [4,5]. Furthermore, recent work considers MFCs to be potential sensoric devices that can measure (i) the quantity or either quality of a biomasses can be monitored and (ii) the appearance of certain toxic compounds, such as heavy metals, so the rate of antibiotic resistance can be determined [6,7,8].

One key component of the above system is the electrogenic bacteria that reside on the anodic surface as the MFC’s operation is based on their metabolism. Efficacy of the system depends on the metabolic activity of the bacteria [9]. In most cases, this is applied in a consortium, i.e., a mixture of several species and strains with diverse metabolic activities.

Recent research on MFC based green energy production has begun to branch in different directions since the application of properly adapted anodic consortia is required to increase efficiency [10]. The research aims to develop specialised consortia that have the ability to break down wastes with either high protein or plant material content, as well as human sewage [11,12]. To fulfil this requirement, different types of electrogenic bacterial consortia that can effectively degrade and utilise biomasses with unique substrate compositions must be established.

Based on the first classical experiments of Benetto [13,14], we know that electron transfer can be based on the presence of mediators, but it may only require close contact between the surfaces of the anode and bacteria. This we call today extracellular electrontransfer (EET). This process can also based on special bacterial surface structures, called nanowires [15,16]. Previous research has also revealed that not only Gram negative, but also Gram positive bacteria were able to carry out EET [17,18]. Today, the number of isolated electroactive bacteria is increasing. Therefore, the number of bacteria that are found to be capable of carrying out direct or indirect electron transfer is also increasing [19,20,21,22,23]. This knowledge broadens the spectrum of known electrogenic bacteria with different metabolic and enzymatic capacities, and based on that knowledge, special microbial consortia can be established that can degrade simple [24,25] or more complex substrates, such as cellulose, lignin or proteins [11,26].

Other applications of MFCs aim to detect certain toxic compounds. Because of proper resolution, this application requires limited numbers of bacteria with well determined toxic compound tolerance range and metabolic activities that are adapted to the environmental niche where the sensoric device will be applied. In these cases, properly sensitive bacteria with special substrate requirements are necessary [27,28,29].

Whether the aim is green energy production or sensoric applications, the capacity of the applied electrogenic microbes to form biofilm on the anodic surface is a requirement along with their special metabolic requirements [24]. Without this feature, microbes are not able to adhere to the anode and EET can not take place.

The aim of this study was to isolate bacteria with electrogenic features from environmental mud samples and examine their potential applications in microbial fuel cell systems. To do so, we characterised their electrogenic features by using two different screening methods, revealed the biofilm-forming capacities of the isolates to different abiotic surfaces, and demonstrated their enzymatic potentials to degrade different macromolecules such as proteins, lipids, and starch. These characterisations will help to establish an anodic consortia for MFC systems that will be able to utilize special biomass substrates.

## 2. Materials and Methods

### 2.1. Mud Sampling and Processing

Bacteria were isolated from mud samples that were taken from six different places along the Hungarian section of Danube River located between Mohács city and the Croatian Hungarian border. Samplings were performed in August 2021, and samples were taken from the mud at a depth of 10 cm. Altogether, 20 samples (between 20–25 g each) were taken and transported to the laboratory for further processing.

### 2.2. Bacterium Isolation and Identification

After homogenisation, one loop from each sample was spread with triple plating onto tryptic soy agar (TSA) (Oxoid, Hampshire, UK) which contained the azo dye Reactive Black 5 (RB5) reagent (Sigma, Germany) [30]. RB5 was mixed from its stock solution in the freshly autoclaved TSA to the final concentration 0.1 g/L. One series of plates were incubated under anaerobic conditions on 23 °C and 30 °C for 3 days, and another series of plates were incubated under aerobic conditions. A purple or yellowish discoloration of the dark blue RB5 around the colonies indicated the electrogenic potential of an isolate. Positive isolates were identified with MALDI-TOF MS (Microflex, Bruker Daltonics). For that purpose, candidate colonies were lifted with a sterile toothpick and spread on the instruments’ sample test plate. For analyses, samples were treated with formic acid and analysed in parallel. Results were compared to the databank.

### 2.3. Tungsten Nanorod Assay for Electroative Bacterium Detection

The ability of the isolates to transfer electrons to electron acceptors located outside the cell was revealed by the tungsten trioxide (WO_3_) nanorod assay, as described earlier [31]. Briefly, 0.825 g of Na_2_WO_4_xH_2_O (Sigma-Merck, Darmstadt, Germany) and 0.290 g of NaCl were dissolved in 20 mL of Milli-Q water. Since the acidic pH is crucial for the nanorod synthesis, the pH was adjusted to 2 by adding HCl solution. The so-gained suspension was transferred into a 100 mL volume Teflon autoclave that was heated to 180 °C in an oven and kept there for 16 h, and than left to slowly cool down. The fluid content of the autoclave was poured out, and the precipitated WO_3_ nanorod was washed thoroughly with deionized water. Finally, it was poured on a ceramic drying plate and completely dried at 60 °C for around 8 h. Prior to the tests, a 5 g/L suspension was made from the collected powder in tryptic soy medium (TSB). From this suspension, 80 microliters were transferred into the holes of a 96-well plate containing 100 µL of bacterium suspension (10^8^ CFU/mL). Each well was layered with 80 μL paraffin oil, and plates were incubated at 23 °C and 30 °C. Colour changes in the wells were monitored 1 h and 24 h after inoculation. Tests were performed in triplicate, and results were evaluated.

### 2.4. Biofilm Assay—96-Well Plate

Biofilm-forming capacities of the isolates were tested using the crystal violet binding plate assay [32]. A total of 20 microliters from the mid-logarithmic phase bacterium cultures were transferred into the wells of a 96-well polystyrene microtiter plate (Sartsedt, Germany) containing 180 μL Luria Bertani (LB) broth. The so-gained suspensions were incubated at 23 °C and 30 °C for 24 h. Planktonic bacteria were removed by gentle washing, and fixed using 2% formaline-PBS solution (Sigma, Germany) for 2 h. After drying, the biofilm layer was stained with 1% crystal violet for 20 min at room temperature. Wells were washed with PBS three times, and the stained layer was solubilised with 200 µL 1% Sodium dodecyl sulphate (SDS) dissolved in 50% ethanol (96%) and 50% PBS. After 2 h, extinction of the solubilized crystal violet solutions were measured at 595 nm in a microplate reader (FLUOstar Optima, BMG Labtech, Aylesbury, UK). Both growth conditions were evaluated in triplicate, and, based on the measured values, were ranked according to a five point scale.

### 2.5. Biofilm Assay—Glass Surface

Examination of the strains’ capacities to adhere and form biofilms on glass surfaces was conducted using a simple test tube method [33] without staining. Mid-log phase bacterium suspensions were diluted 1000× in LB medium, and the so-gained suspension was cultivated in a shaking thermostat (20 rpm) at both 23 °C and 30 °C for 24 h. After incubation, planktonic bacteria were removed by pouring, and the formed biofilm ring structure was visually evaluated and ranked. Both growth conditions were performed in triplicate and ranked according to a five-point scale based on the measured values.

### 2.6. Biofilm Assay on Carbon Tissue

Biofilm-forming capacities of the electrogenic isolates were tested on the conductive PXFT-35 graphite tissue (Zoltek, Hungary). Prior to the experiment, different pretreatment methods were tested to identify the best surface quality that would support biofilm formation. To do so, graphite tissue discs with a diameter of 13 mm were cut with a plug cutter and degreased in 96% ethanol for 30 min. After drying the following treatment combinations were performed: “A”: none; “B”: aceton treatment for 30 min; “C”: 0.1 M NH_3_ treatment for 30 min; “D”: 180 °C heat treatment for 2 h; “E”: aceton treatment for 30 min, followed by 180 °C dry heat treatment for 2 h; “F”: 0.1 M NH_3_ treatment for 30 min, followed by 180 °C heat treatment for 2 h. Prior to the individual tests, biofilm formation was tested in consortium with all the 52 isolates.

The biofilm-formation capacity of each bacterium isolate was tested on the PXFT-35 graphite tissue by using the above combination “E”. The graphite tissue discs with a diameter of 13 mm were cut with a plug cutter and degreased in 96% ethanol for 30 min. After drying, discs were treated with acetone for 30 min and then dry-heat treated at 180 °C for 2 h. Discs were placed into the wells of 24-well tissue culture plates containing 1 mL of 1000× diluted mid-log phase bacterium suspensions. Plates were incubated without shaking at 23 °C for 72 h. The culture medium in the wells was changed every 12 h. After 72 h, incubations discs were prepared for scanning electronic microscopic analyses.

### 2.7. Scanning Electron Microscopy (SEM) Analysis

SEM analyses were carried out as follows [34]. After incubation, medium was pipetted off from each well, and bacteria were adhered to the carbon tissue were fixed with 2.5% glutaraldehyde treatment for 2 h on 4 °C. Fixed bacteria were dehydrated by using 50%, 80%, and 96% gradient ethanol concentrations. Each gradient step was applied for 30 min. After treatment, the carbon tissue discs were dried out. Before obtaining the SEM images, samples were coated with gold with a Jeol JFC-1300 auto fine coater (Jeol, Tokyo, Japan) and surface images were captured with a Jeol JSM-IT500HR (Jeol, Tokyo, Japan) SEM using the secondary electron mode. The accelerating voltage was set to 5 kV and the probe current to 45 kV.

### 2.8. Extracellular Enzymatic Assays

#### 2.8.1. Proteolytic Activity

Ability of the isolates to produce protease was tested with the modified traditional skim milk agar test [35]. First, a 500 mL (2×) basic LB agar solution (5 g yeast extract; 5 g NaCl; 10 g peptone; 15 g agar; 1 L distilled water) was prepared and autoclaved at 121 °C for 20 min. After cooling to 75–80 °C, the 500 mL prewarmed (45–50 °C) 1.5 % skimmed milk was mixed and poured into petri dishes. Plates were incubated at 23 °C and 30 °C, and results were visually evaluated according to the appearance of the clearance zones around the colonies. Positive isolates were ranked according to the sizes and intensities of the zones on a three-point scale. Tests were performed in duplicate at different times.

#### 2.8.2. Lipase Degradation

Tween-80-containing agar plates (15 g peptone; 5 g NaCl; 1 g CaCl_2_; 10 mL Tween 80; 15 g agar; 1 L distilled water) were used to screen the lipase activity of the isolated bacteria [36]. The media was autoclaved, and after solidification, bacteria were streaked on plates. The plates were incubated at 23 °C and 30 °C for 48 h. Appearance of white precipitates around the positive colonies indicated the ability of the isolate to produce lipase. Experiments were performed in duplicate, and activities were ranked on a three-point scale.

#### 2.8.3. Starch Hydrolysis

To test the ability of the isolates to degrade starch, the traditional agar-plate-based assay was carried out [37]. Bacteria were inoculated on starch-agar plates (3 g beef extract; 10 g soluble starch; 15 g agar; 1 L distilled water) and incubated at 23 °C and 30 °C for 48 h. After incubation, the surface of the agar was flooded with Gram’s iodine or Lugol’s iodine solution, and results were recorded immediately. The appearance of zones around the bacterial colonies after the reaction was caused by the production of extracellular enzymes that were able to degrade starch. A lack of zones indicated that the isolate was unable to hydrolyse starch. Experiments were performed in duplicate, and activities were ranked on a three-point scale.

## 3. Results

### 3.1. Bacterium Isolation and Identification

Altogether, 52 bacteria with electrogenic potentials were isolated on the agar plates containing Reactive Black 5 dye that were incubated under either aerobic or anaerobic conditions (Table 1). In several cases, differences among the discoloration intensities of the isolates were revealed under the two conditions. MALDI-TOF MS analyses of the isolates have revealed that the 52 isolates belonged at least to 18 genuses while four isolated species were not identifiable with this method. Among the isolates, representatives of the *Enteribacteriaceae* (such as *Enterococcus* spp., *Citrobacter* spp., *Kelbsiella* sp., *Escherichia* spp., *Enterobacter* sp.), and other families, such as *Aeromonadaceae*, *Shewanellaceae*, *Morganellaceae*, *Pseudomonadaceae*, *Streptococcaceae*, *Staphylococcaceae* were identified.

### 3.2. Tungsten Nanorod Reduction Assay

Further investigation of the electrogenic potentials of the isolates found that most of them had the capacity to reduce the WO_3_ nanorod, which caused the suspension to become bluish (Table 1). In 44 cases (85%), this activity was observable within 24 h, but in the case of 15 isolates (29%), this reaction had already occurred after 1 h (Table 1). Altogether, four isolates did not have the ability to reduce WO_3_ nanorods at all, and only had the capability of reducing RB5.

Furthermore, another four isolates (4, 5, 8, and 15) were able to reduce the WO_3_ nanorod in one hour, but this reducing ability was exhausted on a 24 h timescale; after that, the nanorod was reoxidised and its colour changed back to neutral. Interestingly, this was observed in all of the three *Enterococcus faecalis* as well as the *Carnobacterium divergens* (Table 1).

### 3.3. Biofilm-Forming Capacities on Polystyrene and Glass Surfaces

Marked differences were revealed among the biofilm-forming capacities of the isolates on different surfaces (Table 2). The polystyrene plate with adhesive feature (Plate 83.1835) proved to be best to support the bacterial biofilm formation at either 23 °C or 30 °C. Only isolates 26, 27, and 40, identified as *Enterococcus hirae*, *Salmonella* sp. and *Escherichia coli*, respectively, were unable to form biofilm at 23 °C on this plate. The bacterial-biomass-forming capacity of these three isolates was slightly improved at 30 °C. Meanwhile, for isolate 18 (*S. baltica*), biofilm-formation capacity was also clearly depended on temperature, but it was lower at higher temperature. Interestingly representatives of the same species (isolates 19, 20, 21, 22) showed a much more balanced picture by forming no or minimal biofilms on the two investigated temperatures and on different surfaces.

Generally, biofilm-formation capacity on the nonadhesive plate (Plate 83.1835) was more or less similar to that on the adhesive plate (Plate 83.349), but with lower biofilm-forming intensities.

One group of bacteria showed a fairly balanced picture concerning to their capacities to bind to different surfaces. Isolates 10, 17, 19, 28, 41, 49, and 51 were able to form biofilm to the same extent regardless of whether they were bonded to the adhesive or non-adhesive polystyrene plate or glass surface under different temperatures. There were also fairly weak biofilm formers, such as 26, 33, 40 and 48, which possessed minimal capacities to form a biomass layer that adhered to any of the investigated surfaces. Four isolates (10, 17, 28, 49, and 50) had the capacity to form a very strong biofilm on both plates, on glass tubes, and at both temperatures.

### 3.4. Biofilm-Forming on Carbon Tissue

**Figure 1:**
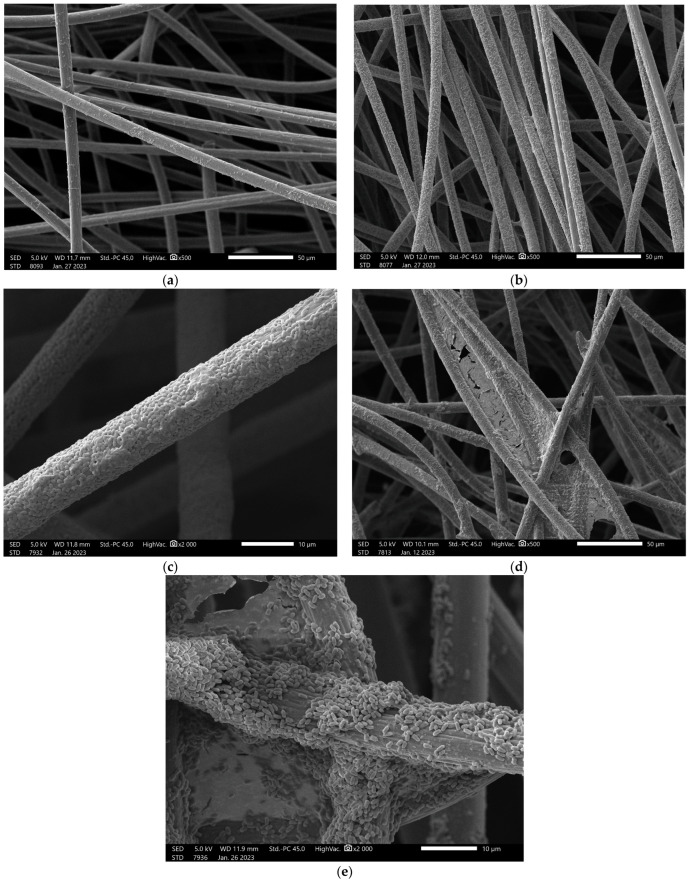
Stages of adhesion and biofilm formation observed on the surfaces of the carbon tissue fibres. (**a**) Presence of sporadic adhered bacteria (+); (**b**) Diffuse, but dense bacterial adhesion (++); (**c**) Confluent monolayer, with multilayer plaques (+++); (**d**) Multilayer biofilm; (**e**) Continuous biofilm also bridging the space between the adjacent fibres. Images of all isolates are summarized in Figure S1.

Almost all mud isolate bacteria could adhere to the surface of the carbon tissue in three days, as was represented on the SEM images (Figure S1). In most cases, however, the adhesion was fairly weak and did not show the typical biofilm structure (Figure S1). These less denser (+) and denser (++) set of bacteria were typically present as monolayers on the surface of carbon fibres (Figure 1). In other cases, bacterial plaques appeared and organised into a multilayer structure (+++). Mature biofilm structures (at least ++++) were visible in eight cases, four of which had a thick structure (+++++) that bridged the space between the adjacent fibres. Thick biofilm structures were formed by the isolates 10, 23, 27, and 36, representing unknown species, *Lelliotittia amnigena*, *Samonella* sp., and a *Buttiauxella ferragutiae*, respectively.

### 3.5. Extracellular Enzymatic Assays

Among the three investigated hydrolytic enzymes, most of the isolates (70%) produced proteases that were detected with a characteristic halo around the colonies cultivated on skimmed-milk agar (Table 3). Lipases were produced to a much lesser extent (38%), and only 29% of the isolates were able to hydrolyse starch with different intensities. A total of 11 isolates had the capacity to degrade all three tested macromolecules at either 23 °C or 30 °C. Temperature dependence was not typical among the isolates, so the intensities were the same at 23 °C and 30 °C.

## 4. Discussion

To develop efficient microbial fuel cell systems with special substrate requirements either for green energy production or for sensoric applications, establishing a proper bacterium consortium is important [11,38,39].

The isolation of 52 bacteria with electrogenic potentials from a natural mud samples demonstrates that electrogenic bacteria can be isolated from all ecosystems and biomass [2,40]. In all the three tables only the genus names were wrote out as based on the MALDI-TOF scores species determinations are not so obvious as in the case of 16 sRNA determinations, that latter one however was not make in this study. The likely bacterium species names were however also added (Table 1).

Interestingly, in the species composition of our collection, *Enetrobacteriaceae* has a dominant presence; this is typically present in animal guts. This could occur because of a slaughterhouse upstream of the sampling locations was releasing contents into the investigated river section. Furthermore, there is a significant amount of cattle and sheep grazing occurring in this flood plain area [41]. *Carnobacterium divergens* and *Aeromonas salmonicida* are associated with fish. *C. divergens* was recently reported as an effective probiotic [42], while the Gram negative *Aeromonas salmonicida* has been reported to be an opportunistic pathogen of fishes that causes septicamia [43]. *Shewanella baltica* was also reported in natural waters and is frequently reported as a spoilage organism in fishes stored at low temperatures [44,45].

Another explanation for the sparse presence of typical environmental isolates could be that, in several cases, they are not easy to cultivate. In several cases, the MALDI-TOF MS databank was unable to identify environmental isolates even at genus level; this was the case with four of our isolates.

Results of the Reactive Black 5 and the WO_3_ nanoreduction assay underline that the isolated bacteria had the capacity to transfer electrons either to RB5, which possesses delocalised electrons, or to the surface of wolfram nanorods (Table 1). Differences in the reducing capacities of the isolates can occur because some of the bacteria may have different EET mechanisms, meaning that only some of them were able to transfer electrons to different acceptors [31]. Our results support this finding as all of our mud isolates were able to reduce the RB5 stain, but only 85% of them had the capacity to reduce the suspended WO_3_ nanorods. This was the case for all three *Enterococcus faecalis* and the only *Carnobacterium divergens* isolate, which is interesting. Exhaustion of the four bacterias’ reducing power could outline an EET mechanism that differs from that of the other isolates which are able to reduce the nanorod, or it could refer to a background mechanism that worked against electron production or accumulation over the long term.

Along with the EET mechanism itself, the quality of the biofilm formed on the anode can influence the electron production and utilisation of a MFC system. Surface qualities, genetic backgrounds, environmental factors, and presence of bacterial adhesins influence this process [46,47]. Because of its practical relevance on many areas of life, several recent studies have focused on the differences in biofilm-forming capacities of bacteria on either biotic or abiotic surfaces [48,49,50,51,52]. Our results are consistent with these previous findings as the biofilm-forming abilities of the mud isolates differed on the 96-well plate and the glass surface (Table 2). However, only slight similarities could be revealed if results were compared to the experiments performed on the carbon tissue. Interestingly, only one biofilm former was able to form a strong biofilm on all the tested surfaces. This unknown species (isolate 10) will be in the focus of further studies.

How biofilm formation effects electron transfer on the anodic surface of an MFC is an open issue. Recent reports have found that damaged biofilm can spoil current production [53], while others have found that several layers of biofilm on the anodic surface could lead to decreased electrochemical performance by increasing the system’s inner resistance [54]. From these and other studies, it can be seen that certain biofilms can support ET while others cannot. We might have thought that low cell densities, which could be observed in “+” and “++” values (Figure 1 and Figure S1) on the surface of the carbon tissue fibres, is not enough to generate electricity, but recent results show that proper current production can be measured despite the low adherence [19].

Beside their electrogenic features, macromolecule degradation abilities of the isolates represented their potential applicability in MFC systems. Isolates with strong protease and lipase activities could be candidates for anodic consortia membership in MFCs that aim to manage waste from the seafood- and dairy industry [39]. The challenge is similar with high-carbohydrate-containing wastes where degradation of complex carbohydrates, such as starch, is preferred [55]. Application of mixed isolates is suggested to be ideal to manage this challenge and increase the efficacy of MFC systems [56]. Overgrown biofilm structures with multispecies compositions, however, are not always ideal for effective electric power harvesting, as the complex structure itself could increase internal resistance in MFC systems [57,58]. Applying a low number of isolates that not only having the capacity to produce a moderate quantity of biofilm, but also consist of bacteria with broad enzymatic spectra or target special wastes may avoid the problem [39]. Considering these aspects, 11 different bacteria (isolates 2, 3, 6, 7, 11, 12, 14, 16, 34, 38, and 47, Table 3), are able to degrade all the three macromolecules and can be ideal candidates for complex waste degradation processes; this means that they can also be good candidates for consortia designed to manage sludge and sewage. Two of them (6 and 7) also have the capacity to form biofilms on carbon tissue, a typical anodic material of MFCs. A similar group can be also identified based on their strong proteolytic activities (4, 5, 26, 40, 44, 45, 48, and 51) in our isolate collection; this could be applied to increase the efficacy of systems that are managing protein-rich wastes [59,60]. Further studies will clarify these aspects, such as how effective will these electrogenic isolates be in real MFC experiments to degrade target macromolecules if applied alone, and how will efficacy be influenced if the isolates are presented in different consortia with different biofilm-forming abilities.

## 5. Conclusions

In this study, 52 mud isolate bacteria were characterised, and their potentials were assessed from different perspectives that could be relevant for MFC applications. Based on the results, idealised anodic consortia with special substrate utilisation capacities can be established and integrated into waste management for different industrial applications. Further studies are required in MFCs for this to be applied.

## Figures and Tables

**Table 1:** Identities and electrogenic potentials of the bacterium isolates collected from mud samples during this study. Electrogenic potentials were revealed on the Reactive Black (RB5) agar plate assay and WO_3_ nanorod reduction assay. (“+” means that they were positive for the test, while “-” means they were negative).

Strain	Genus of Proteins Identified	Growth on LB Agar		Reduction Ability on RB5 Agar		WO_3_ Nanoreduction Assay	
		Aerob	Anaerob	Aerob	Anaerob	1 h	24 h
1	*Enterobacter*	+	+	+	+	-	+
2	*Aeromonas*	+	+	+	+	-	+
3	*Aeromonas*	+	+	+	+	-	+
4	*Enterococcus*	+	+	+	+	+	-
5	*Enterococcus*	+	+	+	+	+	-
6	*Aeromonas*	+	+	+	+	-	+
7	*Aeromonas*	+	+	+	-	-	+
8	*Enterococcus*	+	+	+	+	+	-
9	*unknown*	+	+	+	-	+	+
10	*unknown*	+	+	+	-	-	+
11	*Bacillus*	+	+	+	-	-	+
12	*Aeromonas*	+	+	+	+	-	+
13	*Providencia*	+	+	+	-	-	+
14	*Aeromonas*	+	+	+	-	-	+
15	*Carnobacteriium*	+	+	+	+	+	-
16	*Aeromonas*	+	+	+	-	-	+
17	*Citrobacter*	+	+	+	+	-	+
18	*Shewanella*	+	+	+	+	+	+
19	*Shewanella*	+	+	+	+	+	+
20	*Shewanella*	+	+	+	+	+	+
21	*Shewanella*	+	+	+	+	+	+
22	*Shewanella*	+	+	+	+	+	+
23	*Lelliotittia*	+	+	+	+	-	+
24	*Enterococcus*	+	+	+	+	-	+
25	*Staphylococcus*	+	+	+	-	-	+
26	*Enterococus*	+	+	+	+	+	+
27	*Salmonella*	+	+	+	+	+	+
28	*Escherichia*	+	+	+	+	-	+
29	*Salmonella*	+	+	+	+	-	+
30	*Klebsiella*	+	+	+	+	-	+
31	*unknown*	+	+	+	-	-	+
32	*unknown*	+	+	+	-	-	-
33	*Pleisomonas*	+	+	+	+	-	+
34	*Aeromonas*	+	+	+	+	+	+
35	*Aeromonas*	+	+	+	+	-	+
36	*Buttiauxella*	+	+	-	+	-	+
37	*Citrobacter*	+	+	-	+	-	+
38	*Aeromonas*	+	+	-	+	+	+
39	*Enterococcus*	+	+	-	+	-	+
40	*Escherichia*	+	+	-	+	-	+
41	*Citrobacter*	+	+	-	+	-	+
42	*Lactococcus*	+	+	-	+	-	-
43	*Citrobacter*	+	+	-	+	-	+
44	*Citrobacter*	+	+	-	+	+	+
45	*Citrobacter*	+	+	-	+	-	+
46	*Lactococcus*	+	+	-	+	-	-
47	*Aeromonas*	+	+	+	+	-	+
48	*Lactococcus*	+	+	+	+	-	-
49	*Citrobacter*	+	+	-	+	-	+
50	*Citrobacter*	+	+	-	+	-	+
51	*Lactococcus*	+	+	+	+	-	+
52	*Pantotea*	+	+	+	-	-	+

**Table 2:** Biofilm-forming capacities of the mud-isolated bacteria, tested on adhesive- and nonadhesive polystyrene 96-well plates, glass surfaces, and carbon tissue (PXFT-35). Biomass was ranked on 5-point scale. In case of the 96-well plates the intensities of the solubilised stains, while in case of the glass surfaces, the biofilm ring was ranked. Biofilm-forming capacities of the isolates on the carbon tissue were ranked on a 5-point scale. (+: single and slightly diffuse bacteria can be seen on the surface of the carbon fibres; ++: dense and diffuse bacteria can be seen on the carbon fibres; +++: groups of bacteria can be found on the fibres and some bacterial aggregates (small biofilm initiatives) also appear; ++++: coherent biofilms can be found on the surface of the carbon fibres; +++++: thick and coherent biofilms can be seen on the surface of the fibres and among them.).

Strain	Genus of Proteins Identified	Plate 83.349		Plate 83.1835		Glass Tube		Carbon Tissue
		23 °C	30 °C	23 °C	30 °C	23 °C	30 °C	23 °C
1	*Enterobacter kobel*	-	-	+++	+	+++	+	+
2	*Aeromonas*	-	-	+	-	+	++	++
3	*Aeromonas*	-	-	++	++	+	++	++
4	*Enterococcus*	-	+	++	+	+	+	++++
5	*Enterococcus*	+	-	+	++	-	-	+++
6	*Aeromonas*	+	-	++	+++	++	-	+++
7	*Aeromonas*	+	-	++	+	-	-	+++
8	*Enterococcus*	+++	++	++++	+++	-	-	++
9	*unknown*	+++	+++	+++	+++	+	+	+++
10	*unknown*	+++++	++++	+++++	+++++	+++	+++	+++++
11	*Bacillus*	+++	-	++	++++	+++	++	++
12	*Aeromonas*	-	-	+++	++	+	++	++
13	*Providencia*	++	+	+	+	-	+	+++
14	*Aeromonas*	+++	+++	++	++	-	+	++
15	*Carnobacteriium*	++	-	++	+++	+	+	++
16	*Aeromonas*	++	-	++++	+++	+	++	++
17	*Citrobacter*	+++++	+++	+++++	+++++	+++	+++	++++
18	*Shewanella*	+++++	-	+++++	+	+++	+	+
19	*Shewanella*	+++	++	+++	++	+++	++	++
20	*Shewanella*	-	+	++	+	++	+++	+++
21	*Shewanella*	++	++	++	+	-	+	++
22	*Shewanella*	++	-	+	+	++	+++	+
23	*Lelliotittia*	++	+	+	+	-	++	+++++
24	*Enterococcus*	-	+	+	+	-	+	++
25	*Staphylococcus*	++	++	+++	++++	+	+	+
26	*Enterococus*	+	-	-	+	-	+	-
27	*Salmonella*	-	+	-	+	+++	++	+++++
28	*Escherichia*	+++++	++++	+++++	+++++	++	+++	++
29	*Salmonella*	-	++	+	+++++	+++	+++	++
30	*Klebsiella*	+++	+++	+++	++++	+	++	++
31	*unknown*	++	++	+++	++++	++	++	+++
32	*unknown*	+	+	++	+	-	+	+
33	*Pleisomonas*	-	-	+	+	-	-	++
34	*Aeromonas*	-	-	+	+	++	++	+
35	*Aeromonas*	+++	+	++	++	-	+	++
36	*Buttiauxella*	+++	-	+++	+	+	-	+++++
37	*Citrobacter*	+	-	++	+	++	++	+++
38	*Aeromonas*	++++	+++	+++++	+++++	+	++	+++
39	*Enterococcus*	+++++	-	++++	++++	+	++	++
40	*Escherichia*	-	-	-	+	-	+	++++
41	*Citrobacter*	++	+++	++	+++	+++	+++	++
42	*Lactococcus*	+++	+	+++++	+++++	-	-	++
43	*Citrobacter*	+++	+	++++	+++++	-	+	+++
44	*Citrobacter*	+	+	++++	+++	+++	+	++
45	*Citrobacter*	++	+	++	+	+	++	+
46	*Lactococcus*	+	+	++	+	-	-	++
47	*Aeromonas*	-	+	+	+	+	++	++
48	*Lactococcus*	-	+	+	+	-	-	++++
49	*Citrobacter*	+++++	+++	+++++	++++	+++	++	+++
50	*Citrobacter*	++++	++++	+++	++++	++	+++	
51	*Lactococcus*	++	+	++	++++	+	-	
52	*Pantotea*	+	++++	+	+	+++	+++	

**Table 3:** Capacity of the isolates to produce macromolecule-degrading enzymes, such as protease, lipase, and amylase. (“+++”: strong activity; “++”: medium activity; “+”: low activity; “-”: no activity).

Strain	Genus of Proteins Identified	Protease		Lipase		Amylase	
		23 °C	30 °C	23 °C	30 °C	23 °C	30 °C
1	*Enterobacter*	-	-	-	-	-	-
2	*Aeromonas*	+	+	+++	+++	+++	+++
3	*Aeromonas*	+	+	+++	+++	+++	+++
4	*Enterococcus*	+++	+++	-	-	-	-
5	*Enterococcus*	+++	+++	-	-	-	-
6	*Aeromonas*	+++	+++	+++	+++	+++	+++
7	*Aeromonas*	++	+++	+++	+++	+++	+++
8	*Enterococcus*	++	++	-	-	-	-
9	*unknown*	-	-	-	++	-	-
10	*unknown*	+++	+++	-	-	+++	+++
11	*Bacillus*	+++	++	+	+	+++	++
12	*Aeromonas*	+++	+++	+++	+++	+++	+++
13	*Providencia*	+++	+++	-	-	-	-
14	*Aeromonas*	+++	+++	+++	+++	+++	+++
15	*Carnobacteriium*	-	-	-	-	-	-
16	*Aeromonas*	++	++	+++	+++	+++	+++
17	*Citrobacter*	-	-	-	-	-	-
18	*Shewanella*	-	-	+	+	-	-
19	*Shewanella*	-	-	+	+	-	-
20	*Shewanella*	+	+	+	+	-	-
21	*Shewanella*	-	-	+	+	-	-
22	*Shewanella*	+	+	++	++	-	-
23	*Lelliotittia*	-	-	-	-	-	-
24	*Enterococcus*	++	++	-	-	-	-
25	*Staphylococcus*	++	++	+	+	-	-
26	*Enterococus*	+++	+++	-	-	-	-
27	*Salmonella*	-	-	-	-	-	-
28	*Escherichia*	-	-	-	-	-	-
29	*Salmonella*	-	-	-	-	-	-
30	*Klebsiella*	+	+	-	-	-	-
31	*unknown*	+	+	++	++	-	-
32	*unknown*	-	-	+	-	+	++
33	*Pleisomonas*	-	-	-	-	-	-
34	*Aeromonas*	+++	+++	+++	+++	+	+
35	*Aeromonas*	-	-	+++	+++	-	++
36	*Buttiauxella*	+	+	-	-	-	-
37	*Citrobacter*	++	++	-	-	-	-
38	*Aeromonas*	+++	+++	+++	+++	++	+
39	*Enterococcus*	+++	+++	-	-	+++	++
40	*Escherichia*	+++	+++	-	-	-	-
41	*Citrobacter*	++	++	-	-	-	-
42	*Lactococcus*	++	+++	-	-	-	-
43	*Citrobacter*	-	-	-	-	-	-
44	*Citrobacter*	+++	+++	-	-	-	-
45	*Citrobacter*	+++	+++	-	-	-	-
46	*Lactococcus*	+++	+++	-	-	-	-
47	*Aeromonas*	+++	+++	+++	+++	+++	+++
48	*Lactococcus*	+++	+++	-	-	-	-
49	*Citrobacter*	++	++	-	-	-	-
50	*Citrobacter*	++	++	-	-	-	-
51	*Lactococcus*	+++	+++	-	-	-	-
52	*Pantotea*	-	-	-	-	-	-

## Data Availability

All data are available in this manuscript.

## Supplementary Materials

The following supporting information can be downloaded at: https://www.mdpi.com/article/10.3390/microorganisms11030781/s1, Figure S1: SEM images of the biofilm-forming capacities of the mud isolate bacteria on the pretreated PXFT-35 carbon tissue on 23 °C after 3 days incubation.
