# Supplementary material for: Isolation and Characterisation of Electrogenic Bacteria from Mud Samples
Source: Microorganisms. 2023 Mar 17;11(3):781. doi: 10.3390/microorganisms11030781 (PMC10058994; doi:10.3390/microorganisms11030781)

Supplementary Figure S1.

SEM images of the biofilm forming capacities of each mud isolate bacteria on the pretreated PXFT-35 carbon tissue on 23 °C after 3 days incubation.

1. *Enterobacter koebel*

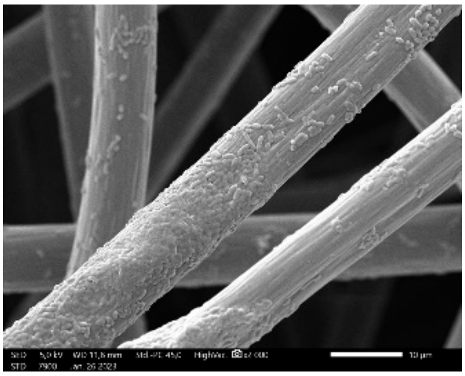

2. *Aeromonas salmonicida*

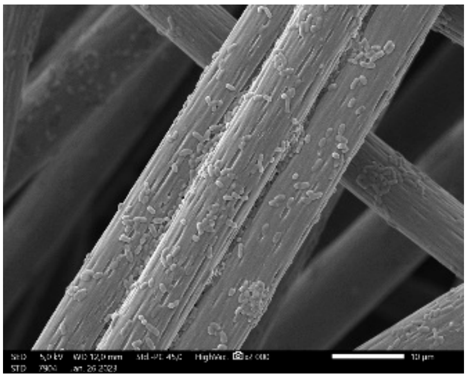

3. *Aeromonas salmonicida*

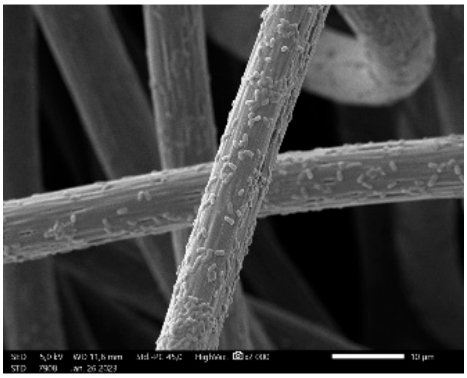

4. *Enterococcus faecalis*

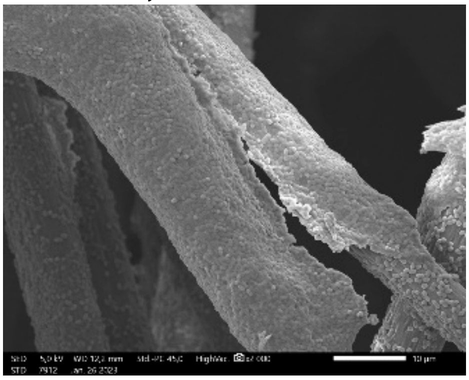

5. *Enterococcus faecalis*

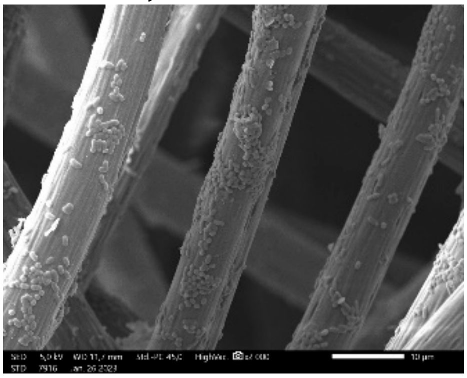

6. *Aeromonas salmonicida*

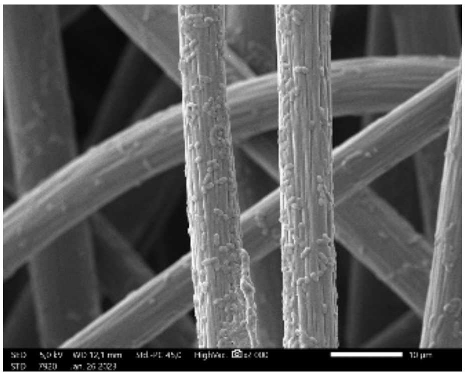

7. *Aeromonas hydrophila*

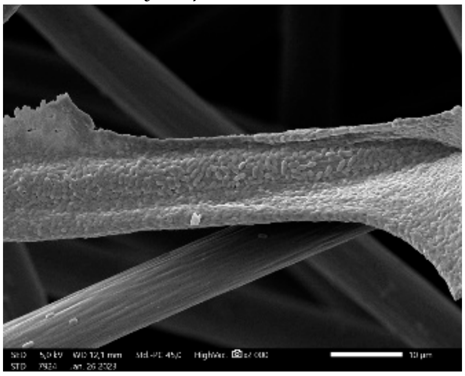

8. *Enterococcus faecalis*

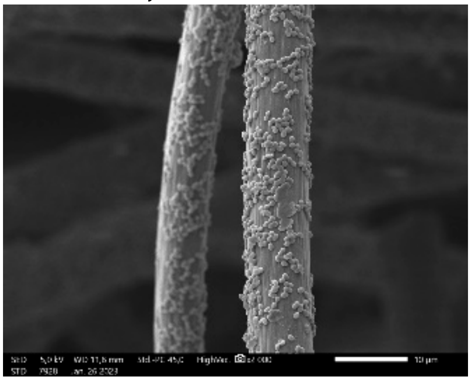

9. *unknown*

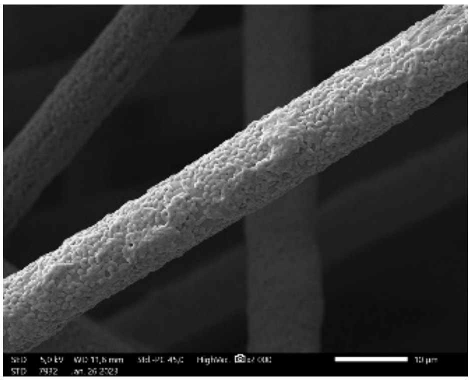

10. *unknown*

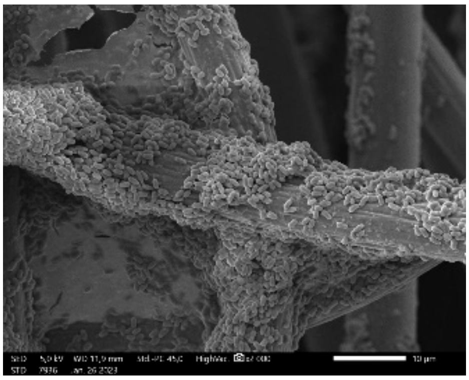

11. *Bacillus cereus*

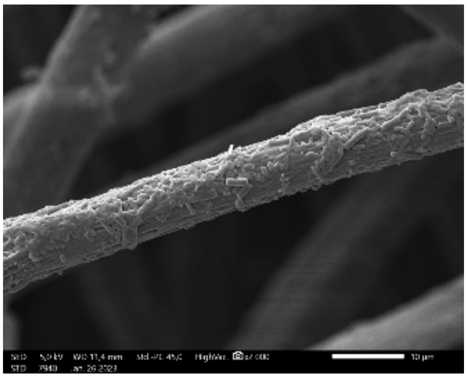

12. *Aeromonas salmonicida*

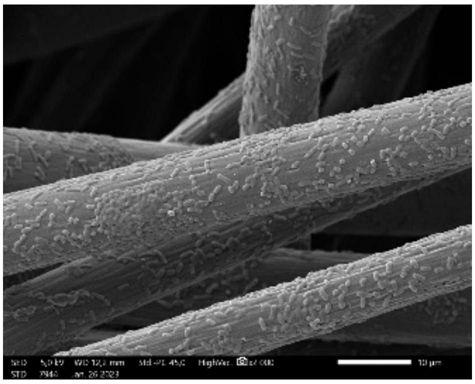

13. *Providencia alkalifaciens*

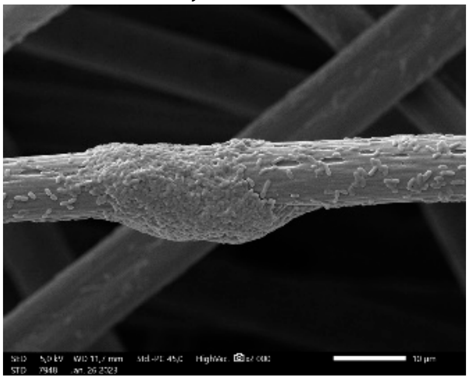

14. *Aeromonas salmonicida*

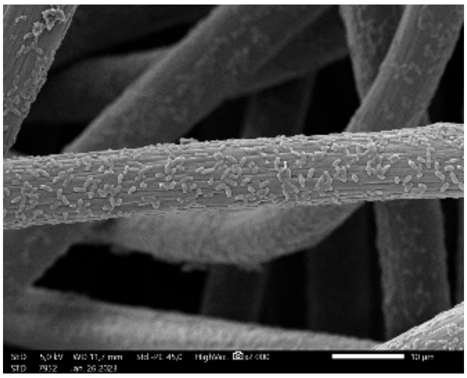

15. *Carnobacterium divergens*

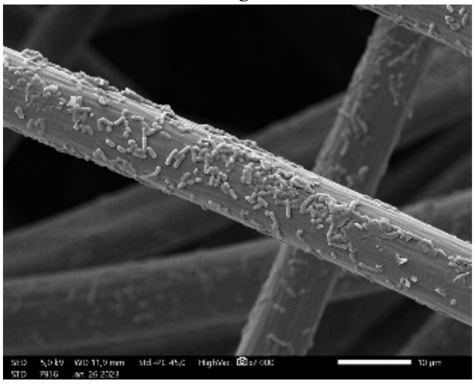

16. *Aeromonas ichtiosoma*

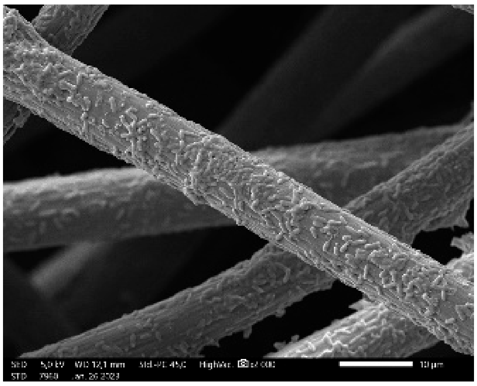

17. *Citrobacter braakii*

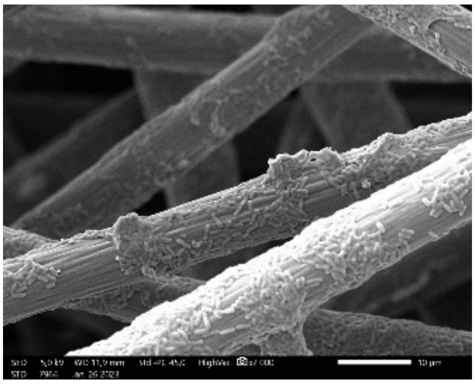

18. *Shewanella baltica*

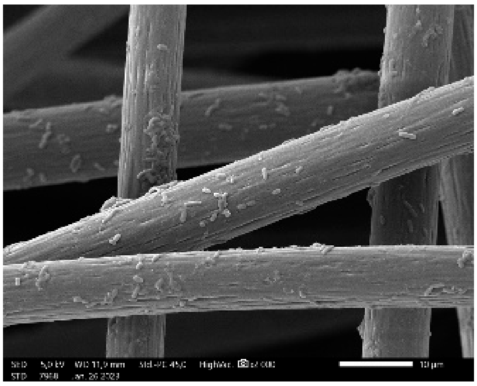

19. *Shewanella baltica*

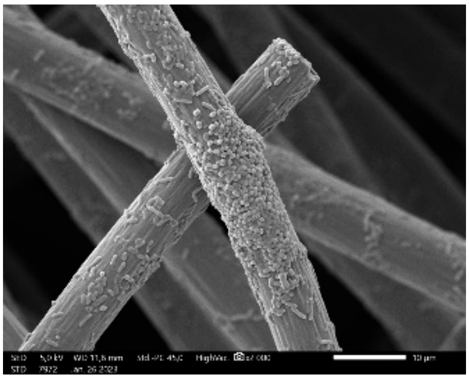

20. *Shewanella baltica*

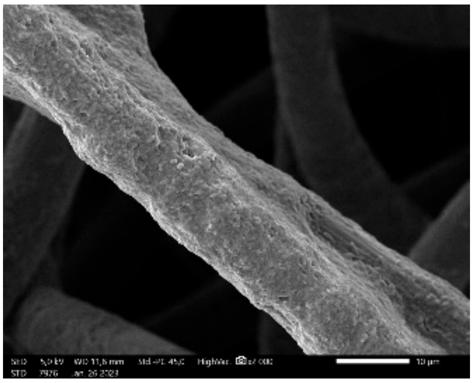

21. *Shewanella baltica*

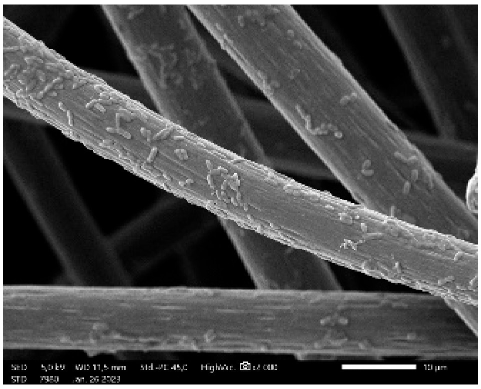

22. *Shewanella baltica*

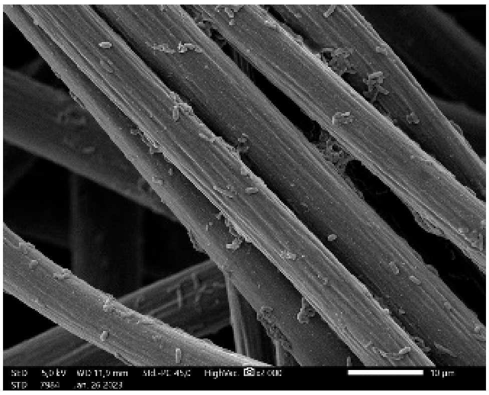

23. *Lelliotittia amnigena*

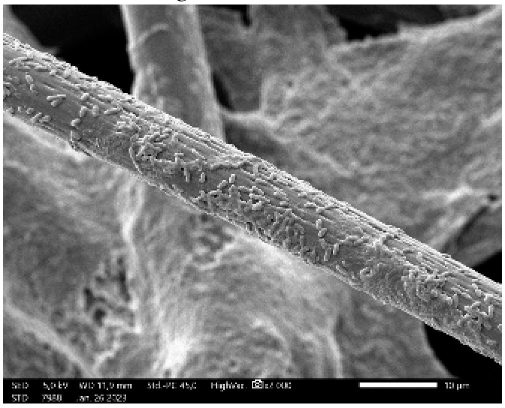

24. *Enterococcus faecium*

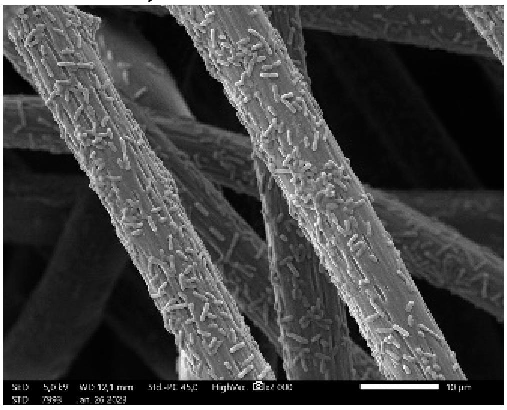

25. *Staphylococcus epidermidis*

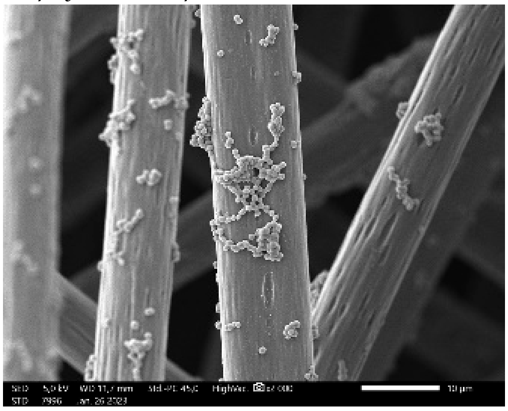

26. *Enterococcus hirae*

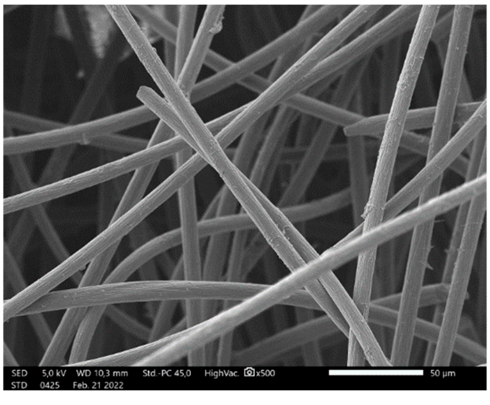

27. *Salmonella sp.*

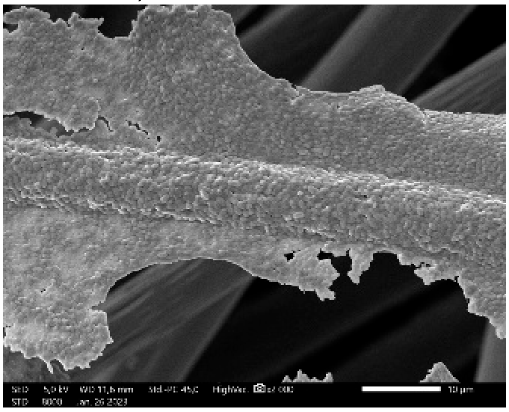

28. *Escherichia coli*

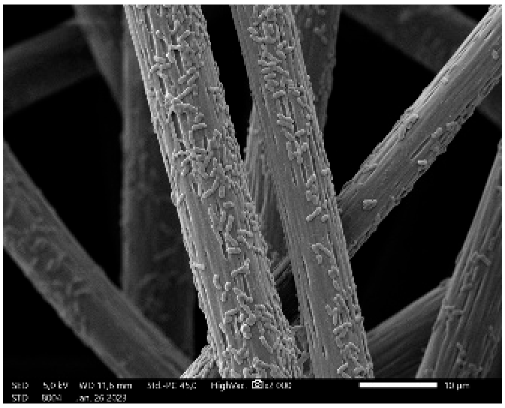

29. *Salmonella sp.*

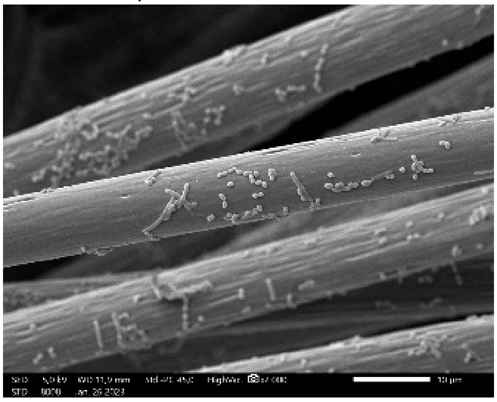

30. *Klebsiella pneumoniae*

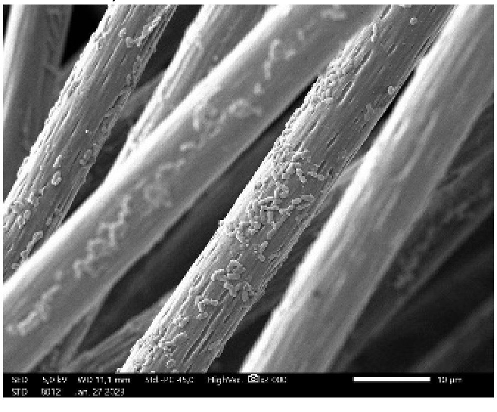

31. *unknown*

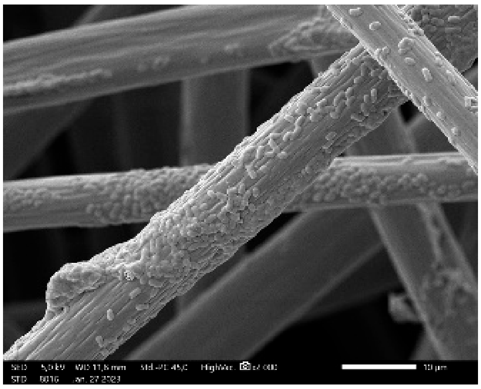

32. *unknown*

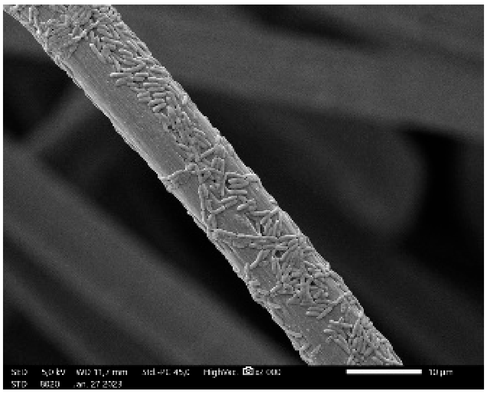

33. *Pleisomonas shigelloides*

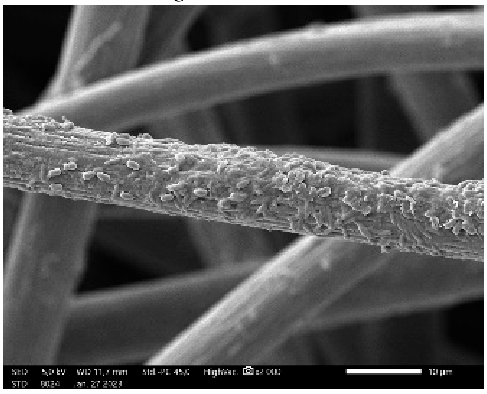

34. *Aeromonas ichtiosmia*

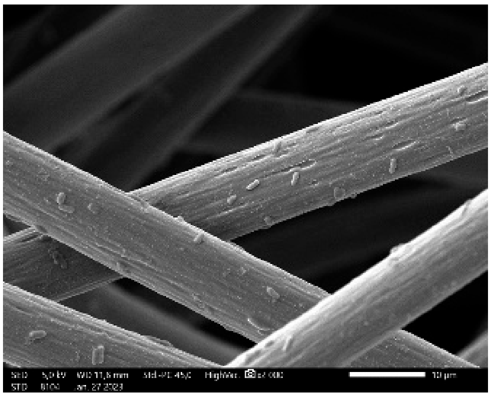

35. *Aeromonas veronii*

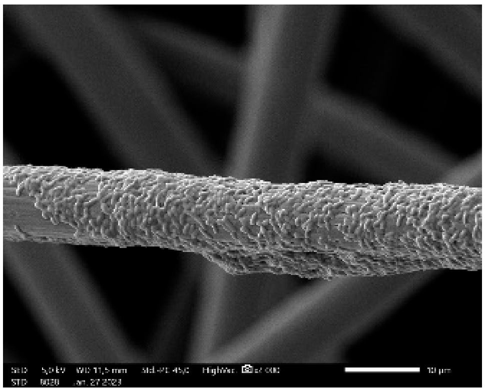

36. *Buttiauxella ferraguitiae*

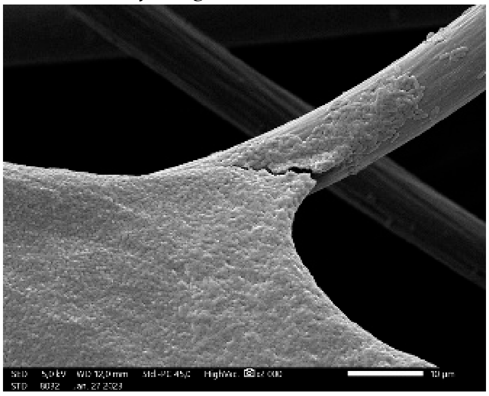

37. *Citrobacter brakkii*

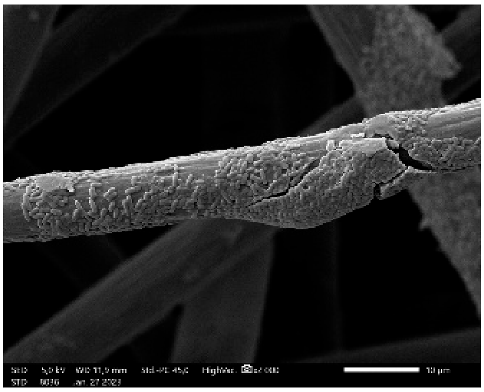

38. *Aeromonas veronii*

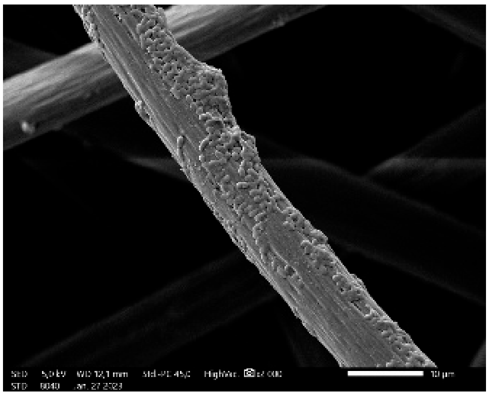

39. *Enterococcus faecium*

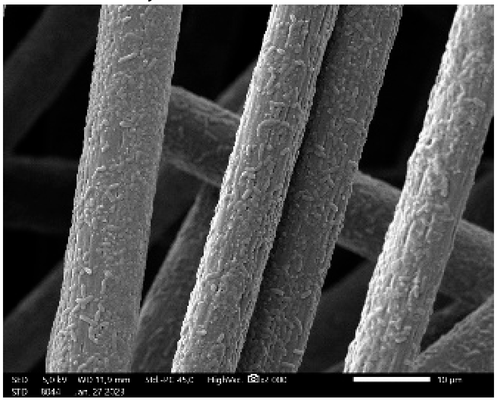

40. *E. coli*

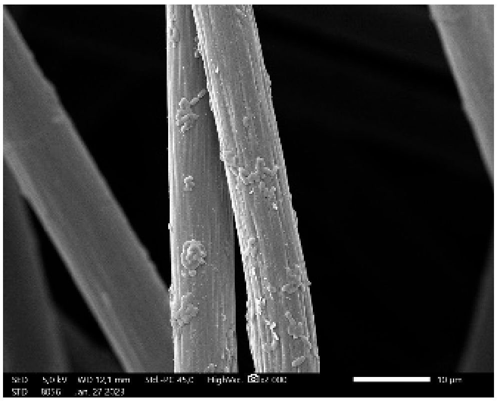

41. *Citrobacter brakkii*

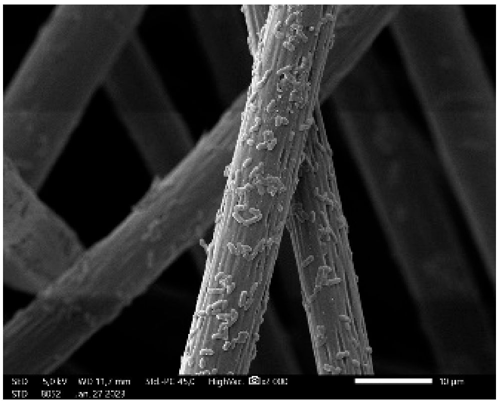

42. *Lacobacillus lactis*

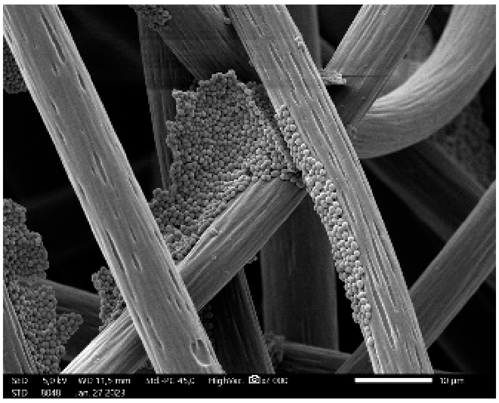

43. *Citrobacter freundii*

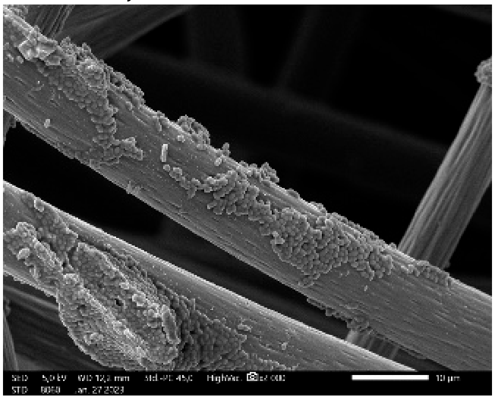

44. *Citrobacter freundii*

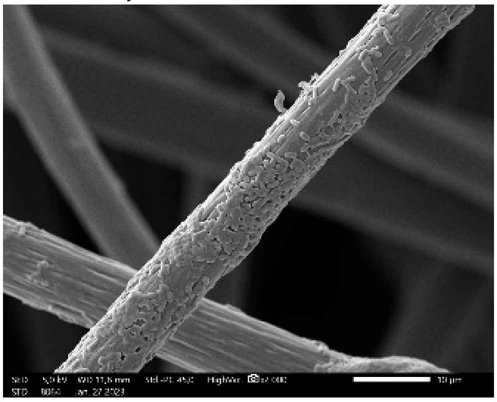

45. *Citrobacter gillenii*

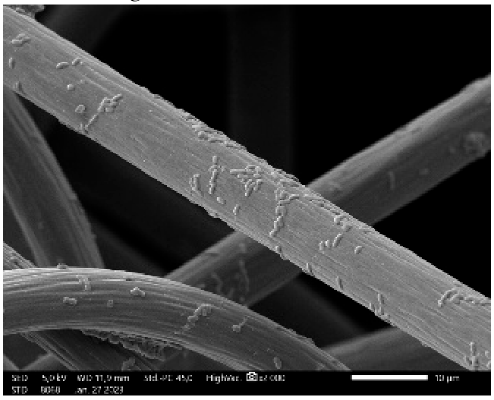

46. *Lactococcus lactis*

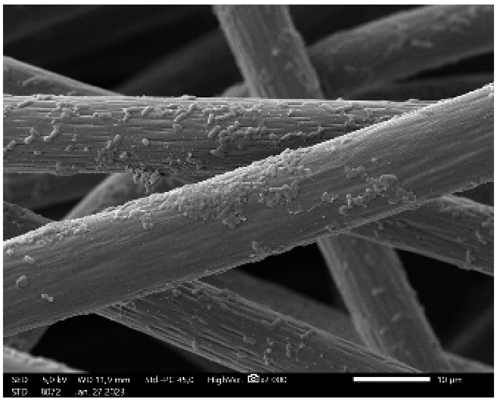

47. *Aeromonas veronii*

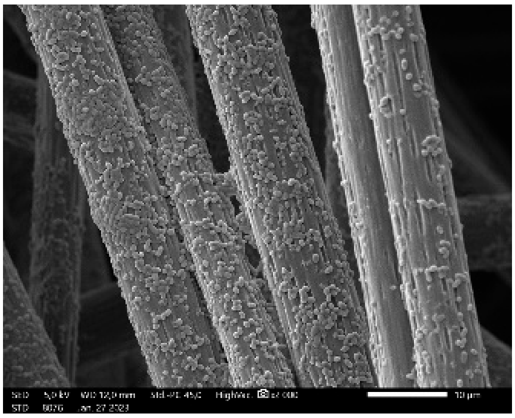

48. *Lactococcus lactis*

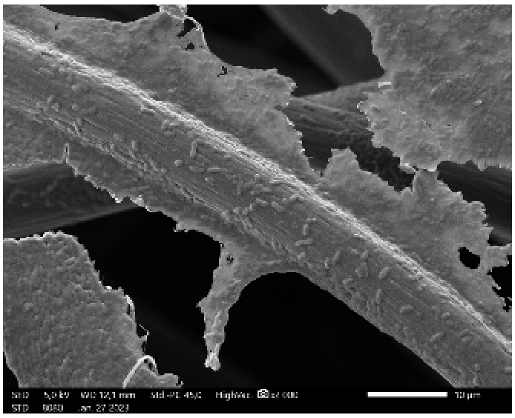

49. *Citrobacter freundii*

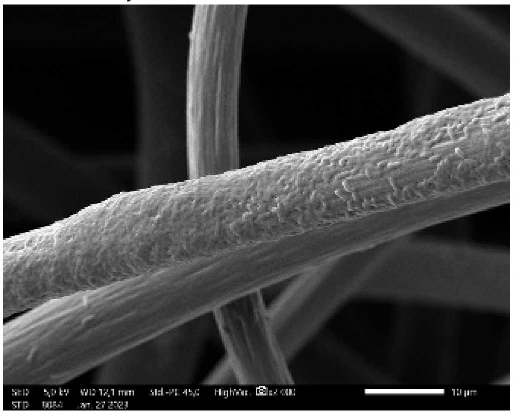

50. *Citrobacter braakii*

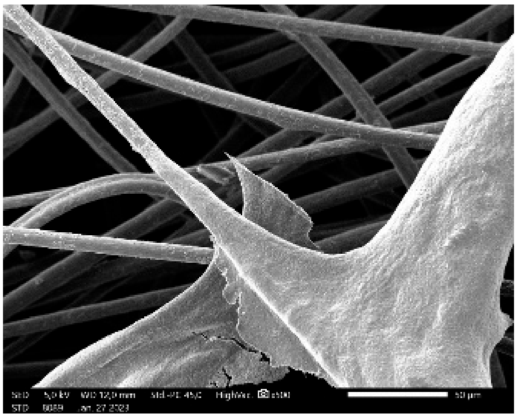

51. *Lactococcus lactis*

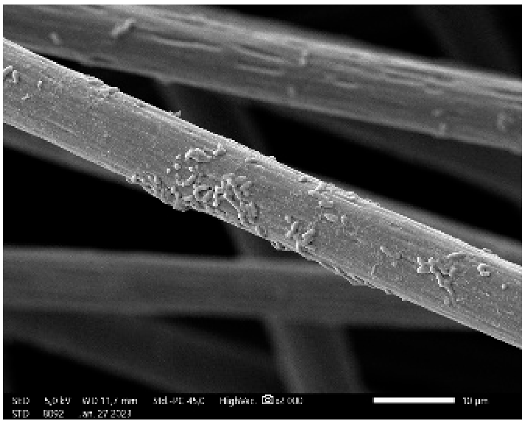

52. *Pantotea agglomerans*

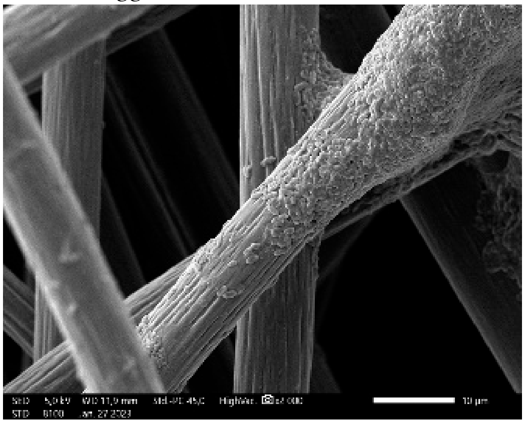

Supplement: Supplementary file 1 [file microorganisms-11-00781-s001.zip › Figure S1.pdf]
